# Combined association of fitness and central adiposity with health-related quality of life in healthy Men: a cross-sectional study

**DOI:** 10.1186/s12955-015-0385-3

**Published:** 2015-11-24

**Authors:** Robert A. Sloan, Susumu S. Sawada, Corby K. Martin, Benjamin Haaland

**Affiliations:** Kagoshima University Graduate School of Medical and Dental Sciences, Kagoshima, Japan; Department of Health Promotion and Exercise, National Institute of Health and Nutrition, Tokyo, Japan; Pennington Biomedical Research Center, Baton Rouge, Louisiana USA; H.Milton Stewart School of Industrial and Systems Engineering, Georgia Institute of Technology, Atlanta, Georgia USA

## Abstract

**Background:**

There is limited data examining the association of combined fitness and central obesity with health related quality of life (HRQoL) in adults. We examined the association of combined cardiorespiratory fitness (CRF) and waist-to-height ratio (WHtR) in the form of a fit-fat index (FFI) with the Physical Component Summary (PCS) and Mental Component Summary (MCS) HRQoL scores in United States Navy servicemen.

**Methods:**

As part of a health fitness assessment, a total of 709 healthy males aged 18–49 years completed a submaximal exercise test, WHtR measurement, and HRQoL survey (SF-12v2) between 2004 and 2006. FFI level was classified into thirds with the lowest FFI tertile serving as the referent group. PCS and MCS scores ≥50 were taken to indicate average or better. Logistic regression was used to obtain odds ratios (OR) and 95 % confidence intervals (CI).

**Results:**

The prevalence of average or better HRQoL scores was lowest in the referent FFI tertile, PCS 60.2 % and MCS 57.6 %. Compared with the lowest FFI group in multivariate analyses, the OR (95 % CI) of having average or better PCS was 1.63 (1.09–2.42) and 3.12 (1.95–4.99) for moderate and high FFI groups respectively; MCS was 1.70 (1.13–2.55) and 4.89 (3.03–7.89) for moderate and high FFI groups respectively (all *P* < 0.001). Consistent and progressive independent associations were observed between age and MCS, and also between CRF and MCS.

**Conclusion:**

Among males in the United States Navy, higher levels of FFI were independently and more consistently associated with having average or better HRQoL (physical and mental) than other known predictors of HRQoL.

## Background

The World Health Organization defines health as “a state of complete physical, mental, and social wellbeing and not merely the absence of disease or infirmity [1]”. In accordance with this definition, the U.S. Centers for Disease Control and Prevention considers health-related quality of life (HRQoL) to be one of the best indicators of an individual’s or group’s perceived physical and mental health over time [2]. People are now living longer due to improvements in health care and consequently there has been increased interest regarding HRQoL [2]. As part of *Healthy People 2020* the U.S. Office of Disease Prevention and Health Promotion has set a key goal for ~80 % of the Nation to have good or better HRQoL by 2020 [3]; concomitantly, the U.S. Armed Forces considers mental and physical functional status to be a critical factor affecting job performance [4]. Previous observational studies reported independent and joint associations between physical activity and measures of adiposity with HRQoL [5–13] but a limitation across studies was the likelihood of bias owing to self-reported physical activity [14] or body mass index (BMI) [15]. To more accurately test associations with HRQoL, some authors advocated for future research to include objectively measured BMI, fat distribution and fitness [12, 13]. Notably, very little is known about objective physical fitness measures in healthy populations with regard to HRQoL. Therefore identifying *practical* and *objective* physical fitness measures related to maximizing HRQoL are needed to better support health promotion and prevention initiatives.

Two objective physical fitness measures that have been independently associated with various health outcomes, including HRQoL, are cardiorespiratory fitness (CRF) and waist-to-height ratio (WHtR). CRF is a metabolic measure of the capacity of the cardiorespiratory system to take up and use oxygen, which can be modified through factors such as physical activity, smoking, body weight, and health status [16]. According to a recent meta-analysis, CRF is one of the strongest independent predictors of all-cause mortality and cardiovascular disease mortality regardless of BMI [17]. While the relationship of fitness and fatness with mortality is well established, less is known in regards to quality of life. Studies have demonstrated CRF to be independently associated with physical HRQoL in clinical and healthy populations [18–23]. However, only one study in a healthy population has demonstrated an association between CRF and mental HRQoL [20]. WHtR is an anthropometric proxy of visceral adiposity. A recent review article discussed the nexus of visceral adiposity with lifestyle behaviors and provided evidence for pathways between visceral adiposity and sleep quality, stress, energy balance, diet quality, and exercise [24]. Unlike BMI, percent body fat, and waist circumference; WHtR allows for a uniform risk value (≥0.50) that is less likely to misclassify adiposity or muscularity in men because of ethnicity or phenotype (i.e., normal weight central adiposity and overweight normal central adiposity) [4, 25]. Furthermore, recent systematic reviews and meta-analyses have demonstrated WHtR to be a better predictor of cardiometabolic health outcomes, diabetes, and mortality than other anthropometric adiposity indicators [25–27]. Two recent studies investigated the relationship of WHtR with HRQoL; one found an independent association between WHtR and mental HRQoL when controlling for lifestyle factors but not when accounting for the existence of any chronic condition [7], while the other study revealed that a ~12 % variation in physical HRQoL could be attributed to changes in WHtR [28]. Controlled trials have also investigated the relative contributions of laboratory measured CRF and adiposity in older, obese and chronically diseased adults [21, 22, 29, 30] and found that small to moderate improvements in one or both measures were associated with higher physical HRQoL scores, with markedly concomitant improvements appearing to influence physical HRQoL more so.

While the findings of the related literature provide some evidence for the independent, joint and relative associations for various indices of fitness and fatness with HRQoL, the combined association of objectively measured fitness and central adiposity with HRQoL in healthy adults is unknown. Therefore, the aim of this study was to examine the association between combined CRF and WHtR in the form of a fit-fat index (FFI) with the probability of having *average or better* physical and mental HRQoL in a sample of healthy U.S. Navy servicemen.

## Methods

### Data source

Data were obtained from the U.S. Naval Hospital Yokosuka, Japan, Health Promotion Center health fitness assessment (HFA) database. A component of the HFA was the Short Form 12 version 2 (SF-12v2™) questionnaire [31]. Trained U.S. Navy medical personnel recorded the data during each HFA. The HFA de-identified data for this observational study was approved as exempt research by the Navy Medical Research Center, San Diego Institutional Review Board.

### Participants

Secondary data were examined from the review of 1127 HFA records of United States Navy male service members age 17–54 years, who were self-referred or referred by their primary care manager for an HFA from 2004 to 2006. The inclusion criteria for the study population sample were young adult males (18–49 years), been in the U.S. Navy for at least six months and graduated high school (*n* = 912). To meet the definition of *healthy*, exclusion criteria included the existence of any chronic condition/disease (*n* = 37), prescribed psychotropic, cholesterol or blood pressure medication (*n* = 124) evidenced by electronic medical records. Members were also excluded based on local center guidelines if they had a pretest elevated resting blood pressure (>160/100 mmHg systolic/diastolic), smoked the morning of the HFA or had an inability to reach 85 % of age-predicted maximal heart rate during the submaximal graded exercise test (*n* = 23). Potential participants were also excluded if variables required for analyses were missing (*n* = 19). After review for inclusion and exclusion criteria, 709 healthy young service members (63 %) of the original database were eligible for analysis. The SF-12v2™ was completed along with a generic self-report health risk appraisal that included tobacco and alcohol use questions.

## Measurements

### HRQoL

HRQoL is defined as the perception of overall satisfaction with life and involves the measurement of functional status in the domains of physical, cognitive, emotional, and social health, and is a fundamental assessment in understanding the health status of an individual or population [2]. The SF-12v2™ is a generic health status instrument that incorporates two HRQoL domains summarized via norm-based scoring algorithms referred to as the Physical Component Summary (PCS) and Mental Component Summary (MCS). Scores ≥50 indicate average or better PCS and MCS respectively when compared to the general population or vice versa [32]. The MCS includes role limitations caused by emotional problems, vitality, social functioning, and mental health and PCS includes physical function, role limitations caused by physical problems, bodily pain, and general health [31, 32].

### Cardiorespiratory fitness

American College of Sports Medicine (ACSM) guidelines for submaximal exercise testing were followed throughout the course of each test and all tests were administered by ACSM certified personnel [33]. Contraindications were determined, medications verified, and appropriate vital signs were taken prior to exercise testing. All members in the data set completed a modified Balke exercise treadmill test starting at 3.3 mph that began with a grade of 0 % and after every 3 min the grade was increased by 3 % until the participant reached 85 % of their age-predicted heart rate max (220-age). The estimated maximal METs of task were calculated by using the method of extrapolation to the age predicted maximal heart rate [33].

### Anthropometric measures

Height and weight were measured with light clothing and without shoes on a calibrated weight scale with stadiometer. BMI was calculated as weight (kg) divided by height squared (m^2^). Waist circumference was measured using the National Institute of Health protocol [34] taken at the top of the right iliac crest with a non-elastic tape measure to the nearest 0.1 cm. WHtR was calculated by dividing waist in cm by height in cm.

### Fit-Fat index

Fit-fat index (FFI) represents the combination of CRF as the estimated maximal metabolic equivalent (MET) and WHtR (MET÷WHtR) expressed in the form of a quotient. Higher scores are considered better and generally range from ~10 to 50 on a continuous scale. FFI compares individuals beyond independent or joint categories for CRF and WHtR (i.e., Unfit/lean, 9.0 METs ÷ 0.45 WHtR = 20; Fit/lean 10.0 METs ÷ 0.49 WHtR = 20; Fit/Fat 11.0 METs ÷ 0.55 WHtR = 20).

### Smoking status and alcohol intake

Participants were questioned in reference to current smoking status (yes or no) and heavy alcohol consumption (>14 vs. ≤ 14 drinks/week).

### Systolic blood pressure

Prior to exercise testing resting blood pressure was measured using the Seventh Report of the Joint National Committee on Prevention, Detection, Evaluation, and Treatment of High Blood Pressure guidelines [35].

## Statistical analysis

Baseline characteristics of participants were compared across tertiles of the FFI using one-way ANOVA for continuous variables and *χ*^2^ tests for categorical variables. The impact of potential covariates on average or better PCS and MCS was assessed with multivariate logistic regression models adjusted for known predictors of HRQoL [36]. To partially mitigate the impact of multicollinearity on the estimation of relative contributions to HRQoL within the same model, predictors with (absolute) correlations exceeding 0.7 were not included in the same multivariate model. The relationship between probabilities of average or better PCS or MCS, and FFI in tertiles was tested with multivariate logistic regression models, adjusted for age alone or age, systolic blood pressure, alcohol habits, smoking habits, and potentially the interaction between CRF and WHtR. To assess possible interaction between the impact of CRF and WHtR on PCS and MCS, a multiplicative interaction term (MET * WHtR) was included in the multivariable model and tested using the Wald test. The lowest tertile for FFI served as the referent group. The Statistical Package for Social Science software was used for statistical analysis (SPSS, Inc., Chicago, Illinois, USA). P-values < 0.05 were considered statistically significant and were two-sided.

## Results

Characteristics of subjects are summarized in Table 1. In brief, all potential confounders were individually associated with each FFI tertile, with the exceptions of current smoking and drinking status.

**Table 1:** Baseline characteristics of men according to fit-fat index levels (tertiles)

Characteristics	All men	1st tertile (Low)	2nd tertile (Moderate)	3rd tertile (High)	P value ^a^
n	709	236	236	237	-
Age (years)	31.6 ± 7.4	34.8 ± 6.5	32.4 ± 7.2	27.7 ± 6.6	<0.001
FFI, median	21.7	17.1	21.7	27.0	<0.001
CRF (METmax)	11.4 ± 1.5	9.8 ± 0.9	11.4 ± 0.7	12.9 ± 1.0	<0.001
Waist/Height ratio	0.53 ± 0.07	0.59 ± 0.05	0.53 ± 0.03	0.46 ± 0.04	<0.001
Height (cm)	176.8 ± 8.4	176.2 ± 9.1	176.0 ± 7.5	178.4 ± 8.2	0.002
Waist circumference (cm)	92.8 ± 11.4	103.7 ± 8.2	92.6 ± 6.3	82.3 ± 7.6	<0.001
Body mass index (kg/m^2^)	28.7 ± 4.3	32.7 ± 3.4	28.5 ± 2.5	25.0 ± 2.7	<0.001
PCS	52.3 ± 7.3	50.1 ± 7.9	52.5 ± 7.1	54.4 ± 6.2	<0.001
MCS	51.3 ± 8.2	50.2 ± 8.4	51.2 ± 8.2	52.4 ± 8.1	0.02
Systolic blood pressure (mmHg)	124.1 ± 11.6	126.9 ± 11.8	123.5 ± 11.3	121.9 ± 11.2	<0.001
Current smokers, n (%)	164 (23.1)	55 (23.3)	60 (25.4)	49 (20.7)	0.47
Current drinkers, n (%)	19 (2.7)	8 (3.4)	6 (2.5)	5 (2.1)	0.68

*FFI* fit-fat index, *CRF* cardiorespiratory fitness (METmax), *PCS* physical component summary, *MCS* mental component summary

Data are means ± SD, unless otherwise specified

^a^ one-way ANOVA for continuous variables and χ^2^ test for categorical variables

There was weak evidence of an interaction between the impact of CRF and WHtR on PCS (P for interaction 0.07). By contrast, there was strong evidence of an interaction between the impact of CRF and WHtR on MCS (P for interaction = 0.01). Thus, the interaction term (MET*WHtR) was tested in multivariable models aimed at the independent association between FFI and each of PCS and MCS.

Independent associations between covariates and the PCS and MCS are summarized in Tables 2 and 3. Independent associations between FFI and both PCS and MCS are summarized in Table 4. There was little evidence that PCS had important independent associations, beyond CRF and FFI. The independent association between PCS and CRF was not progressive, with the odds ratios of average or better PCS approximately equal for the second and third tertiles of CRF (Table 2). On the other hand, the independent association between PCS and FFI was consistent, progressive, and notably strong across multivariate models (Table 4). MCS was independently associated with CRF, FFI, age, and systolic blood pressure. The independent association between MCS and both CRF and FFI was consistent, progressive, and notably strong across multivariate models (Tables 3 and 4).

**Table 2:** Adjusted odds ratios of average or better PCS by potential risk factors

Potential risk factors	Participants	Odds ratio	95%CI	*P* value
Age ^a^				
1st tertile (Low)	253	1.00 (Referent)	―	―
2nd tertile (Moderate)	225	1.23	0.80–1.91	0.35
3rd tertile (High)	231	1.07	0.69–1.65	0.78
Cardiorespiratory fitness ^b^				
1st tertile (Low)	236	1.00 (Referent)	―	―
2nd tertile (Moderate)	236	1.75	1.12–2.73	0.02
3rd tertile (High)	237	1.79	0.99–3.26	0.06
Waist/Height ratio ^c^				―
1st tertile (Low)	253	1.00 (Referent)	―	
2nd tertile (Moderate)	217	0.90	0.58–1.42	0.65
3rd tertile (High)	239	0.78	0.47–1.30	0.34
Waist circumference ^c^				
1st tertile (Low)	292	1.00 (Referent)	―	―
2nd tertile (Moderate)	191	0.91	0.58–1.43	0.69
3rd tertile (High)	226	0.66	0.40–1.08	0.10
Body mass index ^c^				
1st tertile (Low)	238	1.00 (Referent)	―	―
2nd tertile (Moderate)	241	1.16	0.72–1.86	0.54
3rd tertile (High)	230	0.69	0.39–1.21	0.19
Systolic blood pressure ^d^				
1st tertile (Low)	270	1.00 (Referent)	―	―
2nd tertile (Moderate)	229	1.24	0.82–1.87	0.31
3rd tertile (High)	210	1.08	0.72–1.63	0.71
Current smokers ^e^				
No	545	1.00 (Referent)	―	―
Yes	164	0.69	0.47–1.01	0.06
Current drinkers ^f^				
< 14 drinks per week	690	1.00 (Referent)	―	―
≥ 14 drinks per week	19	1.77	0.56–5.57	0.32

*PCS* physical component summary, *OR* odds ratio, *CI* confidence interval

^a^Adjusted for fit-fat index, systolic blood pressure, smoking habit, and drinking habit

^b^Adjusted for age, waist/height ratio, systolic blood pressure, smoking habit, and drinking habit

^c^Adjusted for age, cardiorespiratory fitness, systolic blood pressure, smoking habit, and drinking habit

^d^Adjusted for age, fit-fat index, smoking habit, and drinking habit

^e^Adjusted for age, fit-fat index, systolic blood pressure, and drinking habit

^f^Adjusted for age, fit-fat index, systolic blood pressure, and smoking habit

**Table 3:** Adjusted odds ratios of average or better MCS by potential risk factors

Potential risk factors	Participants	Odds ratio	95%CI	*P* value
Age ^a^				
1st tertile (Low)	253	1.00 (Referent)	―	―
2nd tertile (Moderate)	225	2.26	1.51–3.38	<0.001
3rd tertile (High)	231	5.05	3.21–7.94	<0.001
Cardiorespiratory fitness ^b^				
1st tertile (Low)	236	1.00 (Referent)	―	―
2nd tertile (Moderate)	236	1.69	1.08–2.65	0.02
3rd tertile (High)	237	4.34	2.37–7.94	<0.001
Waist/Height ratio ^c^				
1st tertile (Low)	253	1.00 (Referent)	―	―
2nd tertile (Moderate)	217	0.89	0.58–1.37	0.60
3rd tertile (High)	239	0.80	0.49–1.31	0.37
Waist circumference ^c^				
1st tertile (Low)	292	1.00 (Referent)	―	―
2nd tertile (Moderate)	191	0.75	0.49–1.16	0.19
3rd tertile (High)	226	0.82	0.50–1.33	0.41
Body mass index ^c^				
1st tertile (Low)	238	1.00 (Referent)	―	―
2nd tertile (Moderate)	241	0.81	0.52–1.25	0.34
3rd tertile (High)	230	0.85	0.49–1.47	0.55
Systolic blood pressure ^d^				
1st tertile (Low)	270	1.00 (Referent)	―	―
2nd tertile (Moderate)	229	1.35	0.91–1.99	0.13
3rd tertile (High)	210	1.65	1.10–2.49	0.02
Current smokers ^e^				
No	545	1.00 (Referent)	―	―
Yes	164	0.88	0.60–1.29	0.52
Current drinkers ^f^				
< 14 drinks pre week	690	1.00 (Referent)	―	―
≥ 14 drinks per week	19	1.02	0.39–2.67	0.97

*MCS* mental component summary, *OR* odds ratio, *CI* confidence interval

^a^Adjusted for fit-fat index, systolic blood pressure, smoking habit, and drinking habit

^b^Adjusted for age, waist/height ratio, systolic blood pressure, smoking habit, and drinking habit

^c^Adjusted for age, cardiorespiratory fitness, systolic blood pressure, smoking habit, and drinking habit

^d^Adjusted for age, fit-fat index, smoking habit, and drinking habit

^e^Adjusted for age, fit-fat index, systolic blood pressure, and drinking habit

^f^Adjusted for age, fit-fat index, systolic blood pressure, and smoking habit

**Table 4:** Odds ratios of average or better PCS and MCS according to FFI tertiles

	1st tertile Low (referent)	2nd tertile Moderate	3rd tertile High	*P* value
*PCS*				
Average or better PCS prevalence	60.2 %	71.6 %	82.3 %	―
Age-adjusted OR (95 % CI)	1.00	1.64 (1.11–2.42)	2.91(1.84–4.59)	<0.001
Multivariable OR (95 % CI) ^a^	1.00	1.69 (1.14–2.51)	2.96 (1.86–4.01)	<0.001
Multivariable OR (95 % CI) ^b^	1.00	1.63 (1.09–2.42)	3.12 (1.95–4.99)	<0.001
*MCS*				
Average or better MCS prevalence	57.6 %	63.6 %	72.2 %	―
Age-adjusted OR (95 % CI)	1.00	1.70 (1.14–2.53)	4.19 (2.64–6.63)	<0.001
Multivariable OR (95 % CI) ^a^	1.00	1.79 (1.20–2.68)	4.51 (2.82–7.21)	<0.001
Multivariable OR (95 % CI) ^b^	1.00	1.70 (1.13–2.55)	4.89 (3.03–7.89)	<0.001

*PCS* physical component summary, *MCS* mental component summary, *OR* odds ratio, *CI* confidence interval

^a^adjusted for age, systolic blood pressure, alcohol habits, and smoking habits

^b^ adjusted for age, systolic blood pressure, alcohol habits, smoking habits and MET × WtHR

## Discussion

This observational study investigated the associations between FFI and HRQoL in a sizable sample of healthy U.S. Navy servicemen 18–49 years old. The principal finding was that higher levels of FFI demonstrated independent, strong and consistent associations with having an average or better level of HRQoL. Though inconsistent, CRF also demonstrated independent associations with HRQoL. To the best of our knowledge, this is the first study to evaluate the associations between combined objectively measured CRF and WHtR in the form of FFI with HRQoL in healthy men.

Our study is one of two studies that have tested the association of an index score with HRQoL. Hakkinen and colleagues [18] compared a physical fitness index (PFI) that combined CRF and muscular fitness (grip strength, push-ups, sit-ups and repeated squats) in a sample of 727 young male Finnish military reservists. Similar to our FFI findings with HRQoL, higher PFI associated with better HRQoL while no associations were found between BMI and HRQoL. Of the eight mental and physical HRQoL dimensions [31, 32], PFI was independently associated with two physical dimensions, measured BMI with 0 dimensions, CRF with one physical dimension, and morbidity impairment all eight dimensions. The key similarly between the FFI and the PFI indexes was the use of CRF, while the key difference was the use of WHtR and muscular fitness respectively. The major difference between the sample populations was that a third of the Finnish sample had morbidities (pulmonary or heart disease, hypertension, inflammatory joint disease, or musculoskeletal disease) that impaired all dimension of HRQoL, while our sample population had no documented morbidities.

Another somewhat comparable approach to using a score such as FFI is a joint association analysis. Three large population based studies investigated the joint association between self-reported leisure-time physical activity and BMI [9, 12, 13]. A seven-year prospective study on middle-aged working adults in Finland (*N* = 7332) examined six joint categories in associations with average or better PCS and MCS. Markedly the authors found that the high active/overweight and inactive/normal weight categories had the same odds (OR ~1.5) of having less than average PCS when compared to the referent high active/normal weight category [12]. A similar finding was identified in a nationally representative study of Canadian men (*N* = 23,919) 18–44 years, whereby sixteen joint categories were examined in relation to self-rated overall health, a domain of HRQoL. Men that were in the inactive/overweight and high active/obese categories had the same odds (OR ~ 2.6) of having less than good self-rated overall health when compared to the referent high active/healthy weight category and only the moderately and high active/overweight categories were not a risk when compared to the referent [13]. Heath and Brown also demonstrated this type of risk comparison between leisure time physical activity/BMI joint categories in a nationally representative (*N* = 283,562) study on Americans for odds of ≥14 unhealthy days, physical/mental [8]. While these studies indicated that leisure time physical activity seemed to play a more impactful role on HRQoL the influence of BMI could not be ruled out. Beyond the limitations of using self-reported physical activity and BMI [14, 15], the authors of two of the studies suggested that the findings might have been biased by muscular men having been unfittingly classified as overweight due to the limitations of BMI [12, 13]. While we agree, a plausible explanation may also be that because FFI captures the *degree* of CRF for a given *degree* of WHtR, individuals could have similar FFI scores and thus comparable risks but be in different joint categories for fitness and adiposity. While these studies shed light on the joint associations of fitness and adiposity with HRQoL, they also pointed out the limitations of self-reported physical activity and BMI, the issues of BMI sensitivity and the apparent limitation of fitness and adiposity joint analyses compared to FFI.

Although there are no other studies that have investigated the relationship of FFI per se with HRQoL, there are studies that have used objective measures for analyzing the relative relationships of CRF and adiposity in obese, clinical and older adult populations with HRQoL. Bennet and colleagues [22] investigated the influence of CRF and percent body fat in obese older adults on the association of type 2 diabetes with HRQoL. The analysis demonstrated that for every 10 % increase in percent body fat there was a significant decrease of 1.53 PCS points and for every 1.43 MET increase in CRF there was significant increase 1.25 of PCS points. Rejeski and colleagues [19] also found similar patterns between CRF level and BMI categories in older type 2 diabetics with PCS scores. This kind of relationship was further supported in an 18-month lifestyle intervention study on obese adults at risk for type 2 diabetes, the authors found that for every 5-kilogram loss of body weight or 1.43 MET increase in CRF, there was a significant PCS increase of 1.5 or 3.4 points respectively. Moreover, for every concomitant 5 % weight loss and 10 % increase in CRF there was a significant increase of 6.4 PCS points suggesting that independent changes were present, but simultaneous changes were more effective for impacting physical HRQoL [22]. Although the associations with CRF and adiposity with PCS were in line with our findings, none of the related literature demonstrated significant relationships with MCS. The differences may be due in part to the differences in populations, study design, fitness and adiposity indices used or the existence of morbidities. Collectively our findings along with a small body of evidence that used objective measures for CRF and adiposity suggests that improvements in CRF, adiposity, or both are related to the likelihood of enhanced HRQoL. The construct of FFI is supported because higher CRF, lower WHtR, or some degree of both was consistently related to the likelihood of having average or better HRQoL.

### Strengths and limitations

The present study has several strengths. First, CRF, WHtR, and BMI were measured objectively. In comparison with self-report methods of estimated physical activity, CRF is a more objective and valid measure [14]. It has also been found that self-report methods of BMI are influenced by under-reporting for weight and over reporting for height [15] and, as noted earlier, WHtR is arguably a better practical measure than other anthropometric indices [25]. Second, we used a well-established, valid, and reliable measuring tool for HRQoL that utilized norm-based scoring methodology. Norm-based scoring allows for comparison between other studies that evaluate PCS and MCS regardless of the SF version used and avoids the ceiling effect sometimes seen in the eight SF-36 v2™ domains [5, 31]. Also the norm-based scoring method allows for the threshold classification of average or better (≥50) HRQoL, whereas the majority of studies only examined associated improvement in HRQoL scores. The third unique strength of this study was that the participants we observed were medically documented as healthy, removing the confounder of having a chronic condition. Thus providing emphasis on primary prevention and health promotion services to keep healthy people healthy. Notably, Ware et al. [37] showed in population studies that the existence of *any* chronic condition was associated with baseline HRQoL being below average.

The primary limitation of this study is that it was a cross-sectional observational study from which we cannot determine a causal relationship. Although baseline PCS and MCS mean scores were similar to the apparently healthy U.S. general population and the U.S. military [37, 38], generalizability of these results may be limited because this study was conducted only with healthy males 18–49 years old in the U.S. Navy. Third, self or primary care referral may have biased the results of this study, we attempted to control for this by removing those with chronic conditions. Lastly, although objectively measured submaximal exercise testing is a valid and reliable method, it provides an estimate of CRF [39].

While more studies need to be done, the research application of FFI may serve as a way of reframing the fitness or fatness debate [13, 40] by considering the combined influence of FFI related to health outcomes and perhaps a better way of doing categorical fitness/adiposity joint analyses. From a practice point of view, FFI may provide a more motivating way for individuals to gauge themselves because it operates on a continuum rather than using independent or joint category terms. For example, individuals could increase physical activity and decrease WHtR, thus improve FFI without eliciting changes in CRF or BMI and it is also possible for individuals to have a similar FFI but be classified in different independent or joint categories. The nature of FFI allows individuals to use differing degrees and combinations of lifestyle modifications to improve. The tailoring of lifestyle improvement options may lead to better affect regulation and autonomy, thereby increasing the likelihood of achieving better HRQoL [41]. Lastly, the practicability of assessing FFI in individual, workplace, community and military settings makes it a viable option for assessment and health promotion program development [25, 42, 43].

## Conclusions

Although it is well established that aerobically fit individuals generally have lower levels of adiposity, the results of our study suggest that for a given level of CRF having a healthier WHtR or vice versa was associated with a higher likelihood of having average or better HRQoL in healthy adult men. Given that cross-sectional studies of this nature are used to generate hypotheses, future studies should examine other age groups, females and ethnicities along with prospective and clinical designs that consider other health outcomes beyond HRQoL.
